# The role of radiation therapy in bone metastases management

**DOI:** 10.18632/oncotarget.14823

**Published:** 2017-01-26

**Authors:** Francesca De Felice, Andrea Piccioli, Daniela Musio, Vincenzo Tombolini

**Affiliations:** ^1^ Department of Radiological, Oncological and Anatomo-Pathological Sciences, Policlinico Umberto I Sapienza University of Rome, Rome, Italy; ^2^ Oncology Center, Palazzo Baleani, Policlinico Umberto I Sapienza University of Rome, Rome, Italy

**Keywords:** bone metastases, palliative radiotherapy, pain, spinal cord compression, pathologic fractures

## Abstract

Bone metastases represent an important complication of malignant tumours. Despite improvement in surgical techniques and advances in systemic therapies, management of patients with bone metastatic disease remains a powerful cornerstone for the radiation oncologist. The primary goal of radiation therapy is to provide pain relief, preserving patients quality of life.

## INTRODUCTION

Solid tumors frequently metastasize to bone. The exact incidence of bone metastases is difficult to extrapolate, but it has been estimated that approximately 35.000 people per year in Italy will develop bone metastases [1]. Depending on primary tumor site, the incidence of bone metastases varies extensively, with breast, prostate or lung cancer accounting for over 85% of patients with metastatic disease. Bone metastases usually appear after adequate therapy for primary tumor, but in up to 20% of cases they are the presenting symptom at diagnosis.

Bone metastasis are usually associated with a poor prognosis with median survival rates limited to several months. However, year after year, significant progress in systemic and supportive therapies have increased patients’ life expectancy. Therefore, patients with metastatic disease represent a heterogeneous patient group and overall survival depend on both primary tumor site and concomitant presence/absence of visceral metastases. Nowadays, patients who develop oligometastases from prostate and breast cancer have a longer median survival compared to those with bone-only metastatic lung cancer (36 months *versus* 6 months) [2–3].

Independently of clinical outcomes, the vast majority of patients with bone metastases requires an active treatment, due to pain, difficulty with ambulation, pathologic fractures, spinal cord compression, hypercalcemia and neurologic deficits.

Skeletal metastases are the most common localization, occurring principally in lumbar and thoracic spine, pelvis, ribs and long bones of the upper and lower extremity. Certain skeletal sites are pathognomonic of specific primary tumor. For instance, involvement of the scapular is more common with renal primaries, whereas skull metastases develop more frequently from primary breast cancer.

Several variables, including life expectancy, predicted outcomes, disease characteristics and patient preferences must be considered to tailor the best cancer therapy for the individual patient. Radiation therapy (RT) can represent an effective palliative intervention in metastatic disease to maintain and improve patient's quality of life (QoL). The majority of patients will experience pain during their disease course and pain management can significantly improve their QoL. This review provides highlights in effects, clinical efficacy and tolerability of RT delivered by linear accelerators in the management of patients with bone metastases.

## INDICATIONS AND AIMS OF RADIATION THERAPY

The important role of RT in the palliation of bone metastases is well recognized. RT is performed primarily to relieve pain, definitely control a bone affected from metastases and prevent pathologic fractures as well as spinal cord compression. Radioisotopes can be administered for more diffuse bone pain that is not eligible for palliative RT, whereas bisphosphonates are usually considered in case of multiple unpainful skeletal metastases.

The goals of RT are to improve QoL, reduce analgesic requirements and maintain or ameliorate skeletal function. Beneficial effects on pain may necessitate several days to a few weeks, so analgesic medication must be optimized during that interval [4].

The initial patient evaluation should be adequate and accurate, using validated instruments specific for cancer patients in palliative care. It is important to report patient symptom experience, using a pain scale of 0 - 10, in which 0 represents no pain and 10 represents maximum pain [5]. The European Organisation for Research and Treatment of Cancer Quality-of-Life Questionnaire (EORTC QLQ)-C30, as well as his short form EORTC QLQ-C15-PAL, is commonly used to assess QoL in this setting of patients [5–6 ].

Briefly, first-line palliative RT is indicated for symptomatic bone metastases, after the appearance of new painful site or after failure or insufficient effect subsequent to a first irradiation.

## EFFECTS OF RADIATION THERAPY ON BONE METASTASES

Due to the persistence of red bone marrow, the axial skeleton is seeded more than the appendicular skeleton. Bone metastases are mainly associated to an overstimulation of osteoclasts or osteoblasts causing a lytic or blastic lesion, respectively. Both lytic and blastic lesions can cause bone pain. Osteoclast activation contributes to painful osteolytic metastases due to higher rate of pathologic fractures. Bone cancer pain principally depends on three mechanisms [4, 7]. Firstly, tumor cells alter the physiological equilibrium between osteoclasts and osteoblasts, determining structural degradation of the bone. Secondly tumor cells may directly invade nerve root or may increase expression of chemical mediators which stimulate nerve fibers. Lastly, surrounding muscles may spasm, in order to maintain skeleton stability. These conditions can cause discomfort. The beneficial effects of RT on bone pain are mainly related to its capability to produce ossification. Moreover ionizing radiation are able to diminish osteoclasts activation and kill tumor cells [7]. There will therefore be a reduction in tumor volume, preserving discomfort to adjacent nerves. Furthermore, the reported evidence of symptom relief within 24 hours after initial RT suggests that reduction of both inflammatory cells and chemical pain mediators is involved in this rapid reaction [8].

## FRACTIONATION SCHEDULES

The optimal fractionation schedule is still an unresolved issue. In clinical practice, the selection of the fractionation schemes is often influenced by patient characteristics (performance status, compliance to treatment, life expectancy), tumor-related factors (histology of the primary tumor, interval time from primary diagnosis to bone metastases, time of developing pain or neurologic deficits before RT) and logistic issues (treatment duration time, validity of family members assistance, hospital location, cost of therapy). A summary of the main clinical circumstances is provided in Table 1.

**Table 1:** Bone metastases radiotherapy suggestions

Clinical circumstances	Modality	Radiotherapy schedule	
		total dose	fraction
Painful uncomplicated bone metastases	3D-CRT	8 Gy	1
	SBRT	15-24 Gy	1
		18-36 Gy	3-6
Pathologic fractures	3D-CRT	20 Gy	5
		30 Gy	10
Spinal cord compression post decompressive surgery	3D-CRT		
		20 Gy	5
______________________		30 Gy	10
alone		8 Gy	1
Re-irradiation	3D-CRT	8 Gy	1
	SBRT*	10-30 Gy	1-5

*clinical trials

Abbreviations: 3D-CRT: three-dimensional conformal radiation therapy; SBRT: stereotactic body radiation

### Painful uncomplicated bone metastases

Single fraction RT schedule, prescribed to the appropriate target volume, is recommended as the standard of care for the treatment of symptomatic and uncomplicated bone metastases [9].

During the past two decades, multiple randomized controlled trials (RCTs) have demonstrated pain relief equivalency between a single 8 Gray (Gy) fraction and multiple fraction regimens, including 30 Gy in 10 fractions, 24 Gy in 6 fractions and 20 Gy in 5 fractions [10–15]. Recently, based on 25 RCTs, a meta-analysis has been performed to provide substantial evidence for defining the optimal RT fractionation schedule [16]. Retreatment rates to the same anatomic site due to recurrent pain were higher in those who received single fractions compared to fractionated treatment courses (20% *versus* 8%, *p* < 0.00001). Single fraction treatment optimized patient and caregiver convenience and was related with lower acute toxicity, including nausea and vomiting, fatigue, diarrhoea and skin reactions. Single fraction was also associated with a higher pathological fracture and spinal cord compression rates compared to multiple fraction treatment, but this result did not reach statistical significance (odds ratio [OR] 1.10, 95% confidence interval [CI] 0.65 - 1.86, *p* = 0.72 and OR 1.44, 95% CI 0.90 - 2.30, *p* = 0.13, respectively). However, the limitation of this analysis is mostly related to the primary end-points heterogeneity of the RCTs included.

Despite its proven clinical effectiveness and its cost efficiency, worldwide the single fraction regimen is underused into daily practice for palliative RT for painful bone metastases [17]. The reasons proposed for this reticence are mainly related to a lack of experience with large fraction sizes, participation in RCTs and influence of reimbursement [17]. Since target volume as well as patient characteristics and tumor-related factors were not specifically addressed in RCTs, literature evidence does not support the choice of RT regimen based on these considerations [9].

In case of spinal metastases, percutaneous vertebroplasty combined with adjuvant RT seems to be better that RT alone to relief pain, maintain vertebral body stability and improve patients’ QoL [18].

### Pathologic fractures

Although data are limited, a multiple fractionated treatment, including 20 Gy in 5 fractions and 30 Gy in 10 fractions, could be considered appropriate in those patients at risk of pathologic fractures, in order to guarantee a tumor down-staging prior to surgical approach. On the other hand, where the treatment objective is tumor shrinkage and pain control in presence of a bone lesion associated with large soft tissue mass, no dose-fractionation recommendation can be define [9].

### Spinal cord compression

Metastatic spinal cord compression represents a medical emergency. An immediate, aggressive treatment approach is essential to preserve neurologic function and to improve patient's QoL. RT, surgery or combined modality constitutes the management for spinal cord compression. Although, in the past, RT was considered the standard of care, nowadays, due to improvements in neurosurgical techniques, new evidence suggested that direct decompressive surgery plus postoperative RT seems to be superior to RT alone for spinal cord compression caused by metastatic tumor [19]. The typical dose is 20 Gy (4 Gy/fraction) or 30 Gy (3 Gy/fraction). In those patients unfit for surgery, RT alone is the recommended treatment, although the optimal dose and fractionation schedule is not still established. Considering the limited expected survival in most spinal cord compression patients and that the majority of the patients are quite debilitated, a shorter treatment program is highly desirable. Rades et al [20] presented the largest spinal cord compression patient cohort comparing five different RT schedules to investigate the optimal therapy for these patients. Five RT schedules were compared: 8 Gy (sigle fraction), 20 Gy (4 Gy/fraction), 30 Gy (3 Gy/fraction), 37.5 Gy (2.5 Gy/fraction) and 40 Gy (2 Gy/fraction). No significant differences in term of improvement of motor function after RT and acute or late toxicity were found between the five groups. Recurrences occurred more frequently after shorter treatment course than protracted schedules; but patients irradiated with shorter regimens were retreated, although patients submitted to protracted schedules received no further treatment at the time of recurrence. In a randomized trial, Maranzano et al [21] have compared different dose fractionation schedule, in patients with spinal cord compression and life expectancy < 6 months. Patients were randomized to single 8 Gy fraction *versus* multiple fractions. Both regimens were effective with no significant difference in back pain relief (56% *versus* 59%), motor capacity (68% *versus* 71%) and good bladder function (90% *versus* 89%). Authors suggested single fraction RT has been recommended as regimen of choice in clinical practice for patients with spinal cord compression and short expected survival [22].

## RESPONSE TO RADIATION THERAPY

### Response categories

In order to reduce the variation in the measurement of pain - self-reporting pain *versus* radiation oncologist-based - among trials, the International Bone Metastases Consensus Working Party on palliative radiotherapy defines and recommends the following response categories [5]: 1) Complete response: a pain score of 0 at treated site with no concomitant increase in analgesic intake (stable or reducing analgesics in daily oral morphine equivalent [OMED]). 2) Partial response: pain reduction of 2 or more at the treated site on a scale of 0 to 10 scale without analgesic increase, or analgesic reduction of 25% or more from baseline without an increase in pain. 3) Pain progression: increase in pain score of 2 or more above baseline at the treated site with stable OMED, or an increase of 25% or more in OMED compared with baseline with the pain score stable or 1 point above baseline. 4) Intermediate response: any response that is not captured by the complete response, partial response, or pain progression definitions.

### Response to treatment

Considering that single fraction RT is equally effective to multiple fractions for most patients, the overall response percentages is presented. There is no consistent dose-response relationship for pain relief, suggesting that response is mainly mediated by a change in the local bone environment [8]. Globally, the rates of pain relief ranges from 50% to 85%, with up to one-third reporting complete response [23]. Patients receiving RT for bone metastases will experience complete to partial pain relief, typically within 4 weeks after RT. Mean duration of remission is approximately 19 weeks. Patients with breast cancer or prostate cancer have higher response rates, as well as duration of remission, than patients with lung cancer or other primary tumors [24].

## TOXICITY OF RADIATION THERAPY

QoL plays a progressively more important role in the evaluation of overall treatment efficacy. Due to short life expectancy of the vast majority of metastatic patients, acute toxicity is much more clinically relevant than late complications. However, with improvement in systemic therapies, some patients, especially those with oligometastases, have a better prognosis and, thus, are potentially at risk of developing late toxicity.

Toxicity depends mostly on the total dose delivered to the normal tissues adjacent to the target volume. It is predictable and generally self-limiting. No major differences in gastrointestinal disturbances (including nausea, vomiting and diarrhea), itching, skin reactions and tiredness were reported between single fraction or multiple fractions regimens [16].

Side effects are manageable with conservative measures. For instance, for treatment over the abdomen or with large target volumes in the pelvis, it is reasonable to use a prophylactic antiemetic to reduce acute nausea and vomiting.

Pain flare is described as a temporary increase in bone pain at the treated metastatic site, during or shortly after RT completion [25]. Its physiopathology is still unknown. It has been suggested

to arise from the release of biochemical mediators of inflammation or the edema compressing nerves in the area of treatment [26]. Pain flare incidence has been reported only in recent studies, with great variability from 2% to 40% [27–28]. Patients receiving a single 8 Gy fraction are at higher risk of pain flare, whereas patients with steroids prescribed as part of their systemic therapy are less likely to experience it [29]. The use of anti-inflammatory medications can prevent or minimize the risk of pain flare.

## RADIATION THERAPY TECHNIQUES

### Three-dimensional conformal radiation therapy

**Figure 1:**
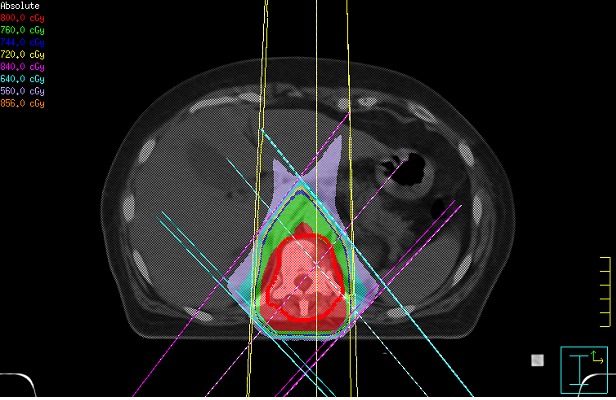
Isodose distribution for three-dimensional conformal radiation therapy (3D-CRT) to dorsal vertebra

Three-dimensional conformal radiation therapy (3-DCRT) is now firmly considered the standard of practice to treat bone metastases. 3-DCRT allows to conform dose distribution to the target volume, reducing dose to the surrounding normal tissues. Before treatment, a computed tomography (CT) scan of the affected anatomic site is obtained. Patient should be positioned in a comfortable and reproducible position. Using a three-dimensional CT planning, the most conformal treatment plan is prepared. The goal is to deliver the maximum dose to the target volume and spare the normal tissues. Figure 1 illustrates treatment of a spinal metastases with single 8 Gy fraction. In vertebral metastases, radiation fields should include the involved vertebral body (and if necessary the soft tissue tumor), plus a vertebral body below and above.

### Stereotactic body radiation

Stereotactic body radiation (SBRT) is a modern treatment modality that delivers high doses to metastatic bone with a great accuracy, minimizing the dose to the adjacent critical structures, primarily spinal cord and cauda equine, but also lung, esophagus, kidney, bowel and contiguous vertebral bodies. Considering the heterogeneity of prognosis for patients with bone metastases, there is a consistent interest to identify a subset of patients with oligometastatic disease - up to three metastatic lesions - who may achieve more durable pain control with SBRT [22, 30].

**Figure 2:**
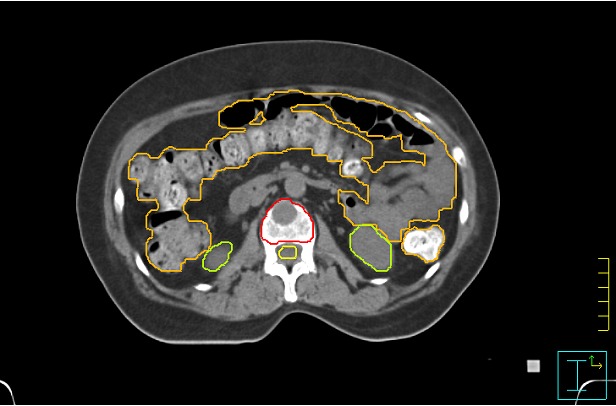
Target volume and organs at risk definition in a stereotactic body radiation therapy (SBRT)

SBRT is performed on vertebral body lesions, without extension to the posterior cortical, allowing for distance between target volume and spinal cord and thus limiting neural structures to threshold below the dose of radiculopathy and myelopathy. SBRT treatment field encompass only the tumor lesion. This equates to the anterior portion of the involved vertebral body, with the inclusion of posterior wall and pedicles in more posterior bone metastases site. A typical target volume definition is shown in Figure 2.

Indications for SBRT include solitary or up to three vertebral metastases. Lesions should be smaller than 5 cm in diameter. Symptomatic spinal cord compression can represent a contraindication to SBRT treatment [31]. Effective patient immobilization, as well as meticulous quality control, are essential during treatment. There is no consensus on dose and fractionation for SBRT. Several fractionation schedules have been proposed from single 15-24 Gy fraction to multiple fractions schedules delivering a total dose of 18-36 Gy. Efficacy and safety data for SBRT are mostly from retrospective single-institution analysis. Generally pain relief and local tumor control were recorded in over 80% of metastatic patients, with an extremely low (less than 0.5%) incidence of myelopathy [32].

## RE-IRRADIATION

Re-irradiation to the same bone site should be considered after initial palliative RT in the following scenario: 1) no response in previously irradiated area; 2) partial response and the hope of additional benefit from repeat treatment; 3) pain relapse after initial satisfactory response.

Patients requiring re-irradiation represent a substantial group, considering that up to 40% of patients do not obtain any pain relief after initial RT and pain relapse occurs in approximately 50% of initial responders within one year after RT [33]. Globally, time to response after re-irradiation varies from 3 to 5 weeks [33]. Patients initially treated with single fraction of 8 Gy are 2.6 times more likely to require re-treatment than those who received multiple fractions [16]. Interestingly, radiation oncologists seem more likely to offer re-treatment after initial 8 Gy RT *versus* initial multiple fractions schedule, may be due to the limits of radiation tolerance [24, 34]. Most multiple fractions patients were not referred to re-treatment due to misconception of high myelopathy risk.

Despite the huge number of patients undergoing re-irradiation, the paucity of data limits definitive guidelines. The International Bone Metastases Consensus Working Party published a consensus on palliative RT in an attempt to promote appropriate set of end-points, including re-irradiation criteria [35]. In the updated version, the Consesus Working Party recommends a 4-week interval for re-treatment in those patients who do not achieve a response to initial RT [5]. The best available evidence on re-treated patients was presented by van der Linden et al [24]. They reanalyzed the database of the Dutch Bone Metastasis Study, the largest study on the effect on painful bone metastases of 8 Gy single fraction *versus* 24 Gy in multiple fractions. The choice to re-irradiate was left to the radiation oncologist's discretion. Mean time to re-treatment was 13 weeks in single fraction patients and 21 weeks in multiple fractions patients. For the different primary tumors, mean time to re-treatment was 22 weeks for prostate cancer, 14 weeks for breast cancer, 11 weeks for lung cancer and 13 weeks for other primary tumors. Apparently, a longer waiting period to start re-irradiation was observed in patients with prostate cancer compared with the other primary tumors. However only 19% of progressive prostate patients responded again. Thus it seems appropriate to no postpone re-irradiation for too long. Regarding the effectiveness of re-treatment, response was recorded in 66% initial 8 Gy patients and 46% initial multi fractions patients (*p* = 0.12), with longer mean duration of remission in initial single fraction patients (16 weeks *versus* 8 weeks).

At present no definitive recommendation can be given regarding dose and fractions in re-irradiation. Single 8 Gy treatment should be considered a valid option. This suggestion is based on the results of the RCT that compared the pain-relieving efficacy of two dose fractionation schedules - single 8 Gy fraction *versus* 20 Gy in multiple fractions - in patients with bone metastases requiring re-treatment [36]. This was a multicentre, non-inferiority trial and a total of 850 patients were randomly assigned to single or multiple fractions re-irradiation. In term of response and efficacy, 8 Gy in a single fraction was non-inferior to 20 Gy administered in multiple fractions. Globally, RT with 8 Gy was less toxic than 20 Gy treatment, whereas QoL, assessed using the EORTC QLQ-C30, was similar between the two treatment regimens.

In case of localized RT-refractory painful bone metastases, SBRT could be considered a treatment option. In previously irradiated bone metastases, several phase I/II trials tested different doses, including 24 Gy in 3 fractions and 10-30 Gy in 1-5 fractions [37–38]. However, specifics of dosing and target volume delineation are still not well-defined and there is no evidence of superiority of SBRT over conventional EBRT with respect to pain control. SBRT should be reserved for patients who are treated in specialized centers and preferably within the confines of a therapeutic clinical trial [23]. Careful patient selection in terms of performance status, number of metastases, primary tumor type, and loco-regional anatomy, is paramount.

## FUTURE PERSPECTIVES

Currently, new emergent targeted therapies blocking key agents for the development and progression of bone metastases have been tested, especially in prostate cancer field [39]. Based on the available literature, several new bone target therapies, including mTOR inhibitors, anti-androgens and radium-223 have shown to improve overall survival in bone metastatic patients, whereas other new molecules, such as anti RANKL/RANK pathway are still under investigation [39]. Targeted therapies represents an attractive therapy that should be added to bone metastatic clinical trial practice but how best to incorporate it with RT remains an urgent need. Data regarding the safety profile of combining RT and targeted therapies in bone metastatic patients are very limited and further investigations are needed [40].

Recently, the combination of RT and ablative approaches, including radiofrequency ablation (RFA), high intensity focused ultrasound (HIFU) and cryoablation has been proposed as therapeutic option to control painful bone metastases [41–42]. But it is too early to establish definitive conclusions. The sequence of treatments, as well as the optimal combinations should have a better understanding.

## SUMMARY

RT is a well-accepted local treatment modality for patients with painful bone metastases. Effective use of RT requires a well consolidate multidisciplinary approach and a close cooperation between surgical, radiation and clinical oncology, as well as nursing personal is paramount.

Single fraction treatment is efficacious and cost-effective for the relief of painful bone metastases.

Additional data are required to define the correct fractionation in spinal cord compression and pathological fractures. SBRT has emerged as a modern high precision technique able to improve pain control with durable effect, especially in retreatment setting or oligometastatic patients. However, further data are paramount to better define its role in this scenario.

## References

[1] Santini D, Lanzetta G (2015). Trattamento delle metastasi ossee. Linea Guida AIOM.

[2] Hayat MJ, Howlader N, Reichman ME, Edwards BK (2007). Cancer statistics, trends, and multiple primary cancer analyses from the Surveillance, Epidemiology, and End Results (SEER) Program. Oncologist.

[3] Kuchuk M, Kuchuk I, Sabri E, Hutton B, Clemons M, Wheatley-Price P (2015). The incidence and clinical impact of bone metastases in non-small cell lung cancer. Lung Cancer.

[4] Lutz S (2012). The role of radiation therapy in controlling painful bone metastases. Curr Pain Headache Rep.

[5] Chow E, Hoskin P, Mitera G, Zeng L, Lutz S, Roos D, Hahn C, van der Linden Y, Hartsell W, Kumar E (2012). International Bone Metastases Consensus Working Party. Update of the international consensus on palliative radiotherapy endpoints for future clinical trials in bone metastases. Int J Radiat Oncol Biol Phys.

[6] Groenvold M, Petersen MA, Aaronson NK, Arraras JI, Blazeby JM, Bottomley A, Fayers PM, de Graeff A, Hammerlid E, Kaasa S, Sprangers MA, Bjorner JB (2006). EORTC Quality of Life Group. The development of the EORTC QLQ-C15-PAL: a shortened questionnaire for cancer patients in palliative care. Eur J Cancer.

[7] Goblirsch MJ, Zwolak PP, Clohisy DR (2006). Biology of bone cancer pain. Clin Cancer Res.

[8] Vakaet LA, Boterberg T (2004). Pain control by ionizing radiation of bone metastasis. Int J Dev Biol.

[9] Wu JS, Wong RK, Lloyd NS, Johnston M, Bezjak A, Whelan T (2004). Supportive Care Guidelines Group of Cancer Care Ontario. Radiotherapy fractionation for the palliation of uncomplicated painful bone metastases - an evidence-based practice guideline. BMC Cancer.

[10] (1999). No authors listed. 8 Gy single fraction radiotherapy for the treatment of metastatic skeletal pain: randomised comparison with a multifraction schedule over 12 months of patient follow-up. Bone Pain Trial Working Party. Radiother Oncol.

[11] Hartsell WF, Scott CB, Bruner DW, Scarantino CW, Ivker RA, Roach M, Suh JH, Demas WF, Movsas B, Petersen IA, Konski AA, Cleeland CS, Janjan NA (2005). Randomized trial of short- versus long-course radiotherapy for palliation of painful bone metastases. J Natl Cancer Inst.

[12] Foro Arnalot P, Fontanals AV, Galcerán JC, Lynd F, Latiesas XS, de Dios NR, Castillejo AR, Bassols ML, Galán JL, Conejo IM, López MA (2008). Randomized clinical trial with two palliative radiotherapy regimens in painful bone metastases: 30 Gy in 10 fractions compared with 8 Gy in single fraction. Radiother Oncol.

[13] Steenland E, Leer JW, van Houwelingen H, Post WJ, van den Hout WB, Kievit J, de Haes H, Martijn H, Oei B, Vonk E, van der Steen-Banasik E, Wiggenraad RG, Hoogenhout J (1999). The effect of a single fraction compared to multiple fractions on painful bone metastases: a global analysis of the Dutch Bone Metastasis Study. Radiother Oncol.

[14] Nielsen OS, Bentzen SM, Sandberg E, Gadeberg CC, Timothy AR (1998). Randomized trial of single dose versus fractionated palliative radiotherapy of bone metastases. Radiother Oncol.

[15] Gaze MN, Kelly CG, Kerr GR, Cull A, Cowie VJ, Gregor A, Howard GC, Rodger A (1997). Pain relief and quality of life following radiotherapy for bone metastases: a randomised trial of two fractionation schedules. Radiother Oncol.

[16] Chow E, Zeng L, Salvo N, Dennis K, Tsao M, Lutz S (2012). Update on the systematic review of palliative radiotherapy trials for bone metastases. Clin Oncol (R Coll Radiol).

[17] Fairchild A, Barnes E, Ghosh S, Ben-Josef E, Roos D, Hartsell W, Holt T, Wu J, Janjan N, Chow E (2009). International patterns of practice in palliative radiotherapy for painful bone metastases: evidence-based practice?. Int J Radiat Oncol Biol Phys.

[18] Cao XY, Liu YS, Lei MX, Liu SB, Zhou SG, Cao YC, Jiang WG (2016). [Comparison of curative effect and prognosis analysis of patients with spinal metastases treated by percutaneous vertebroplasty combined with postoperative radiotherapy and radiotherapy alone]. [Article in Chinese]. Zhonghua Yi Xue Za Zhi.

[19] Patchell RA, Tibbs PA, Regine WF, Payne R, Saris S, Kryscio RJ, Mohiuddin M, Young B (2005). Direct decompressive surgical resection in the treatment of spinal cord compression caused by metastatic cancer: a randomised trial. Lancet.

[20] Rades D, Stalpers LJ, Veninga T, Schulte R, Hoskin PJ, Obralic N, Bajrovic A, Rudat V, Schwarz R, Hulshof MC, Poortmans P, Schild SE (2005). Evaluation of five radiation schedules and prognostic factors for metastatic spinal cord compression. J Clin Oncol.

[21] Maranzano E, Bellavita R, Rossi R, De Angelis V, Frattegiani A, Bagnoli R, Mignogna M, Beneventi S, Lupattelli M, Ponticelli P, Biti GP, Latini P (2005). Short-course versus split-course radiotherapy in metastatic spinal cord compression: results of a phase III, randomized, multicenter trial. J Clin Oncol.

[22] Maranzano E, Trippa F, Casale M, Costantini S, Lupattelli M, Bellavita R, Marafioti L, Pergolizzi S, Santacaterina A, Mignogna M, Silvano G, Fusco V (2009). 8Gy single-dose radiotherapy is effective in metastatic spinal cord compression: results of a phase III randomized multicentre Italian trial. Radiother Oncol.

[23] Lutz S, Berk L, Chang E, Chow E, Hahn C, Hoskin P, Howell D, Konski A, Kachnic L, Lo S, Sahgal A, Silverman L, von Gunten C (2011). Palliative radiotherapy for bone metastases: an ASTRO evidence-based guideline. Int J Radiat Oncol Biol Phys.

[24] van der Linden YM, Lok JJ, Steenland E, Martijn H, van Houwelingen H, Marijnen CA, JW; Leer (2004). Dutch Bone Metastasis Study Group. Single fraction radiotherapy is efficacious: a further analysis of the Dutch Bone Metastasis Study controlling for the influence of retreatment. Int J Radiat Oncol Biol Phys.

[25] McDonald R, Chow E, Rowbottom L, DeAngelis C, Soliman H (2014). Incidence of pain flare in radiation treatment of bone metastases: A literature review. J Bone Oncol.

[26] Gomez-Iturriaga A, Cacicedo J, Navarro A, Morillo V, Willisch P, Carvajal C, Hortelano E, Lopez-Guerra JL, Illescas A, Casquero F, Del Hoyo O, Ciervide R, Irasarri A (2015). Incidence of pain flare following palliative radiotherapy for symptomatic bone metastases: multicenter prospective observational study. BMC Palliat Care.

[27] Chow E, Ling A, Davis L, Panzarella T, Danjoux C (2005). Pain flare following external beam radiotherapy and meaningful change in pain scores in the treatment of bone metastases. Radiother Oncol.

[28] Hird A, Chow E, Zhang L, Wong R, Wu J, Sinclair E, Danjoux C, Tsao M, Barnes E, Loblaw A (2009). Determining the incidence of pain flare following palliative radiotherapy for symptomatic bone metastases: results from three canadian cancer centers. Int J Radiat Oncol Biol Phys.

[29] Loblaw DA, Wu JS, Kirkbride P, Panzarella T, Smith K, Aslanidis J, Warde P (2007). Pain flare in patients with bone metastases after palliative radiotherapy--a nested randomized control trial. Support Care Cancer.

[30] Palma DA, Salama JK, Lo SS, Senan S, Treasure T, Govindan R, Weichselbaum R (2014). The oligometastatic state - separating truth from wishful thinking. Nat Rev Clin Oncol.

[31] Guckenberger M, Sweeney RA, Flickinger JC, Gerszten PC, Kersh R, Sheehan J, Sahgal A (2011). Clinical practice of image-guided spine radiosurgery--results from an international research consortium. Radiat Oncol.

[32] Bhattacharya IS, Hoskin PJ (2015). Stereotactic body radiotherapy for spinal and bone metastases. Clin Oncol (R Coll Radiol).

[33] Huisman M, van den Bosch MA, Wijlemans JW, van Vulpen M, van der Linden YM, Verkooijen HM (2012). Effectiveness of reirradiation for painful bone metastases: a systematic review and meta-analysis. Int J Radiat Oncol Biol Phys.

[34] Johnstone C, Lutz ST (2014). External beam radiotherapy and bone metastases. Ann Palliat Med.

[35] Chow E, Wu JS, Hoskin P, Coia LR, Bentzen SM, Blitzer PH (2002). International consensus on palliative radiotherapy endpoints for future clinical trials in bone metastases. Radiother Oncol.

[36] Chow E, van der Linden YM, Roos D, Hartsell WF, Hoskin P, Wu JS, Brundage MD, Nabid A, Tissing-Tan CJ, Oei B, Babington S, Demas WF, Wilson CF (2014). Single versus multiple fractions of repeat radiation for painful bone metastases: a randomised, controlled, non-inferiority trial. Lancet Oncol.

[37] Sahgal A, Ames C, Chou D, Ma L, Huang K, Xu W, Chin C, Weinberg V, Chuang C, Weinstein P, Larson DA (2009). Stereotactic body radiotherapy is effective salvage therapy for patients with prior radiation of spinal metastases. Int J Radiat Oncol Biol Phys.

[38] Choi CY, Adler JR, Gibbs IC, Chang SD, Jackson PS, Minn AY, Lieberson RE, Soltys SG (2010). Stereotactic radiosurgery for treatment of spinal metastases recurring in close proximity to previously irradiated spinal cord. Int J Radiat Oncol Biol Phys.

[39] Pantano F, Iuliani M, Zoccoli A, Fioramonti M, De Lisi D, Fioroni I, Ribelli G, Santoni M, Vincenzi B, Tonini G, Santini D (2015). Emerging drugs for the treatment of bone metastasis. Expert Opin Emerg Drugs.

[40] https://www.clinicaltrials.gov.

[41] Di Staso M, Gravina GL, Zugaro L, Bonfili P, Gregori L, Franzese P, Marampon F, Vittorini F, Moro R, Tombolini V, Di Cesare E, Masciocchi C (2015). Treatment of Solitary Painful Osseous Metastases with Radiotherapy, Cryoablation or Combined Therapy: Propensity Matching Analysis in 175 Patients. PLoS One.

[42] https://www.clinicaltrials.gov.

